# Institutional proficiency and learning curves in robotic-assisted thoracoscopic surgery: a single-center retrospective analysis using the cumulative sum method

**DOI:** 10.1007/s11701-025-02387-1

**Published:** 2025-05-13

**Authors:** Tsuyoshi Uchida, Hirochika Matsubara, Chihiro Tando, Koshi Mobara, Mamoru Muto, Aya Sugimura, Hiroyuki Nakajima

**Affiliations:** Department of Thoracic Surgery, Yamanashi University, Chuo, Yamanashi Japan

**Keywords:** Robot-assisted thoracoscopic surgery, Learning curve, Skill transfer

## Abstract

Robot-assisted thoracoscopic surgery (RATS) has advanced the field of minimally invasive thoracic surgery. Its learning curve is reportedly shorter than that of video-assisted thoracoscopic surgery. However, few studies have examined how institutional proficiency evolves with the introduction of new surgeons and how this transition impacts surgical outcomes in RATS. This single-center retrospective study, conducted at a university hospital in Japan, included 154 patients who underwent RATS lobectomy between November 2018 and May 2024. The study population consisted of four thoracic surgeons at different stages of RATS experience. Operative metrics and learning curves were evaluated using the cumulative sum method. Trends in operating time, console time, blood loss, and non-console time were analyzed to assess surgeon-specific performance, and complications, if any, were recorded. The mean operating time was 206.5 min, console time was 153.3 min, and mean blood loss was 23.9 g. The lead surgeon demonstrated a typical upward convex learning curve, whereas subsequent surgeons showed smaller peaks. Non-console time increased during transitions between surgeons. Postoperative complications occurred in 13 patients, none of whom required conversion to thoracotomy. Mechanical malfunctions were noted in 11 cases and were resolved without significant delays. This study demonstrated that introducing new surgeons did not compromise institutional proficiency, indicating effective skill transfer. Optimizing training strategies to reduce early inefficiencies remains an important goal. In conclusion, structured training and workflow support may help maintain institutional proficiency during the expansion of RATS programs. Further prospective studies are recommended to validate training models and promote consistent surgical outcomes.

## Introduction

Robot-assisted thoracoscopic surgery (RATS) lobectomy execution has been reported to require a shorter learning curve compared with video-assisted thoracoscopic surgery (VATS), with proficiency generally achieved after 20–28 cases for RATS versus 28–35 cases for VATS [1, 2]. While previous studies have focused on individual surgeon proficiency, the impact of institutional proficiency—the collective ability of a surgical team to maintain quality outcomes as new surgeons are trained—remains unclear.

Surgical training inherently involves a trade-off between educating new surgeons and maintaining high-quality patient care. As new surgeons gain experience, fluctuations in surgical performance—such as prolonged operative times and increased blood loss—can occur, potentially affecting patient outcomes. While hands-on training is essential for developing surgical skills, excessive variability in outcomes can compromise patient safety. Therefore, balancing effective surgeon education with consistent surgical quality is a fundamental challenge in surgical programs [3].

To objectively evaluate learning curves and performance variations, the cumulative sum (CUSUM) method has been widely utilized in medical performance assessment since its introduction in the 1960s [4, 5]. Previous studies using CUSUM have demonstrated that RATS has a shorter learning curve compared to other minimally invasive procedures at the individual level [6]. However, the broader impact of institutional proficiency on surgical outcomes, particularly in the context of team expansion, remains unexplored.

At our institution, RATS was introduced in November 2018, initially performed by a lead surgeon who later trained three additional surgeons. If the expansion of the surgical team negatively impacts patient outcomes, a reassessment of our training approach may be necessary. This study aimed to evaluate institutional proficiency in RATS by examining surgical quality trends as new surgeons join the program, utilizing the CUSUM method to track performance changes. The findings may provide insights into optimizing training strategies while maintaining high standards of surgical care.

## Materials and methods

### Study design

We conducted a retrospective analysis of all robot-assisted lobectomies performed between November 2018 and May 2024. Our institutional review board approved the study protocol and waived the requirement for informed consent from participants.

### Patient demographics, characteristics, and variables

Patient characteristics, including age, sex, lung location, total tumor size, tumor size of the solid component, and tumor stage according to the 8^th^ edition of the tumor, node, metastasis (TNM) staging system, were documented before surgery. Surgical time, console time, non-console time, blood loss, conversion rate, and reasons for conversion were intraoperatively recorded. Postoperative complications, according to the Clavien–Dindo classification, were documented.

### Surgeons’ expertise and training in VATS and RATS anatomical lung resections

To begin performing robot-assisted surgeries, all surgeons received Intuitive Surgical certification, including online Da Vinci technology modules, simulator exercises (> 20 h), and console surgeon training (a 1-day course with hands-on practice).

This study involved four surgeons (A–D). At the initiation of RATS, each surgeon’s experience with VATS was as follows: Surgeon A, 13 years of experience with > 300 anatomical lung resections; Surgeon B, 8 years of experience with 200 anatomical lung resections; Surgeon C, 7 years of experience with 200 anatomical lung resections; Surgeon D, 7 years of experience with 150 anatomical lung resections.

### Surgical technique

The robotic surgeries were performed using the Da Vinci™ Xi surgical system (Intuitive, Sunnyvale, CA, USA/M3 Robotics, Tokyo, Japan). A 0° optic was used for all procedures. Patients were positioned in the lateral decubitus position with lateral thoracic flexion.

**Fig. 1:**
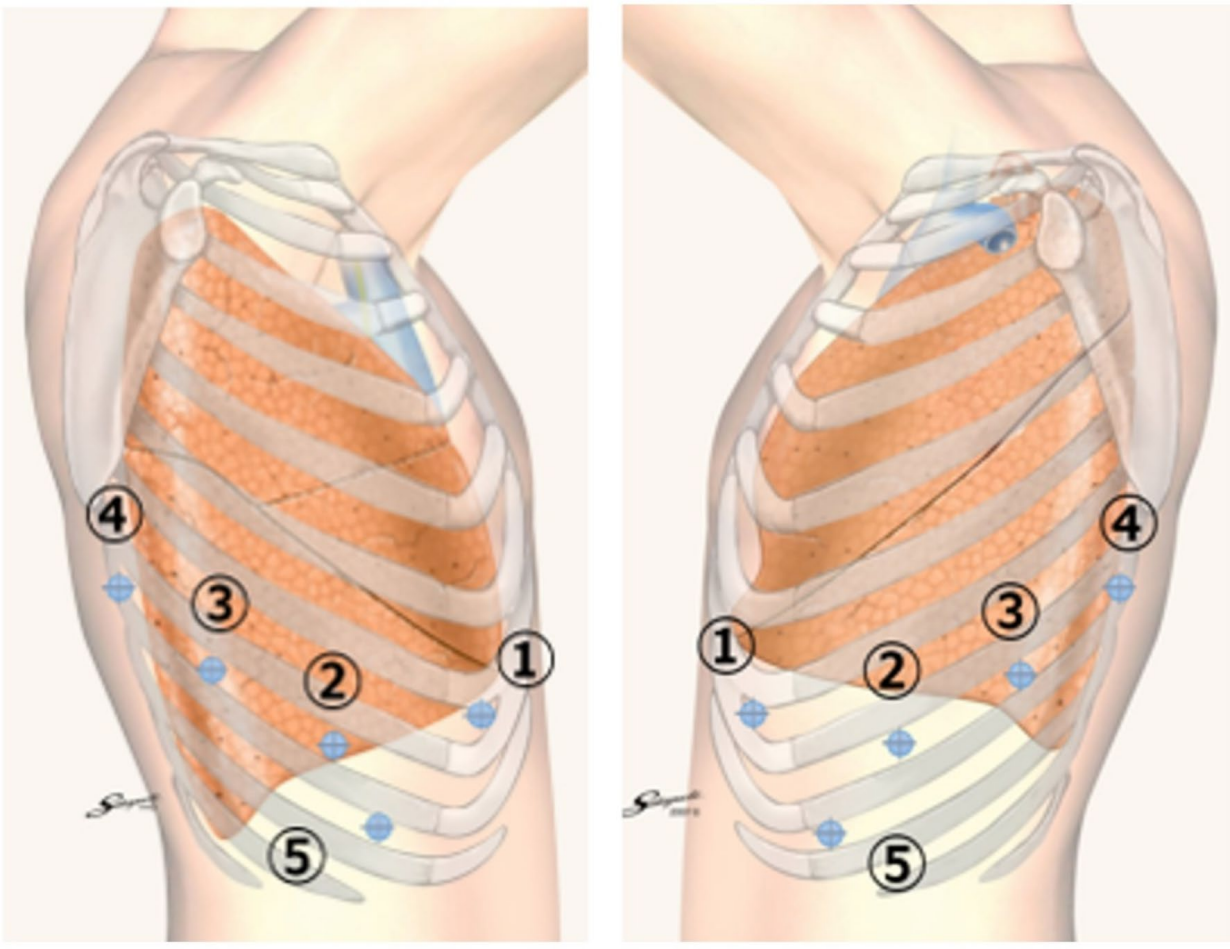
Port placement of robot-assisted thoracoscopic surgery lobectomy. Image used with permission from the Echicon website (https://ess.jjkkpro.jp/material/anatomy02)

Port placements were as follows (Fig. 1):

- Port 1: Seventh intercostal space
- Ports 2–4: Eighth intercostal space
- Port 5: Ninth intercostal space

Carbon dioxide (CO₂) insufflation was applied at low pressure (5–10 cmH₂O) with flow control (flow rate: 5) using the Air Seal™ system (MC Medical, Tokyo, Japan) (Fig. 1).

### Surgical time and console time

Surgical time was defined as the time from skin opening to closure. Console time was defined as the time spent operating the Da Vinci system. Non-console time was defined as the difference between surgical time and console time.

### Conversion

Conversion occurs when a surgical procedure initiated through RATS is later switched to another approach, such as VATS, or open surgery for emergency, technical, or oncological reasons. Regardless of the type of conversion, the final surgery and outcomes were recorded and included in a comprehensive database.

### Analysis of the learning curve

The learning curve was analyzed using the cumulative sum (CUSUM) method to evaluate trends in surgery time, console time, and blood loss over consecutive cases. CUSUM is a statistical tool used to monitor performance changes by calculating the cumulative deviation of individual data points from the mean of a dataset. For each case, the CUSUM statistic was calculated as:

$$\text{CUSUM}(\text{n}) =\text{ CUSUM}(\text{n}-1) + [\text{X}(\text{n})-\text{X}(\text{mean})]$$

where CUSUM(n − 1) is the CUSUM value for the previous case, X(n) is the observed value for the current case, and X(mean) is the reference value.

The reference value (X(mean)) for each measure (surgery time, console time, and blood loss) was determined as the mean of all cases included in the study. This mean was calculated from the entire dataset rather than being derived from a specific subgroup or external benchmark.

A positive trend in the CUSUM chart indicates performance above the mean (e.g., longer surgery times or increased blood loss). In contrast, a negative trend indicates performance below the mean (e.g., shorter surgery times or decreased blood loss). The inflection point in the CUSUM curve is often used to identify where the learning curve stabilizes, reflecting consistent proficiency in the procedure. For the analysis of individual learning curves, the reference value (X(mean)) was defined as the mean operative metric specific to each surgeon, rather than the overall institutional mean.

## Results

### Patient demographics and characteristics

We analyzed 154 lung resections. The median age of participants was 71 (65–77), the majority were male (56.5%), in clinical stage I (90%) and pathological stage I (78.6%), and the right upper lobe was the most commonly treated (46.1%). The median operative time was 196 min (160–242), console time was 145 min (111–180), non-console time was 50 (42–59) min, and blood loss was 10 g (1–19). Patient demographics and characteristics are summarized in Table 1. Surgeon A performed 107 surgeries over 62 months, Surgeon B performed 34 surgeries over 37 months, Surgeon C performed 9 surgeries over 9 months, and Surgeon D performed 4 surgeries over 3 months.

**Table 1:** Results detailing patient demographics and surgical parameters

Variable		
Age, years, median (IQR)		71 (65–77)
Sex		
Female/male, n (%)		67 (43.5)/87
Treated lobe		
LUL, n (%)		28 (18.1)
LLL, n (%)		19 (12.3)
RUL, n (%)		71 (46.1)
RML, n (%)		10 (6.5)
RLL, n (%)		26 (16.9)
Clinical stage TNM 8th		
0, n (%)		7 (4.5)
I, n (%)		138 (90.0)
II, n (%)		7 (4.5)
III, n (%)		1 (0.6)
IV, n (%)		1 (0.6)
Pathological stage TNM 8th		
0, n (%)		6 (3.9)
I, n (%)		121 (78.6)
II, n (%)		20 (13.0)
III, n (%)		2 (1.3)
IV, n (%)		2 (1.3)
Another tissue, n (%)		3 (1.9)
Brinkman index		
Median (IQR)		460 (0–829)
Operative time, min, median (IQR)		196 (160–242)
Console time, min, median (IQR)		145 (111–180)
Non-console time, min, median (IQR)		50 (42–59)
Blood loss, gram, median (IQR)		10 (1–19)

*LUL* left upper lobe, *LLL* left lower lobe, *RUL* Right upper lobe, *RML* right middle lobe, *RLL* right lower lobe, *IQR* interquartile range, *TNM* tumor node metastasis

### Learning curve

**Fig. 2:**
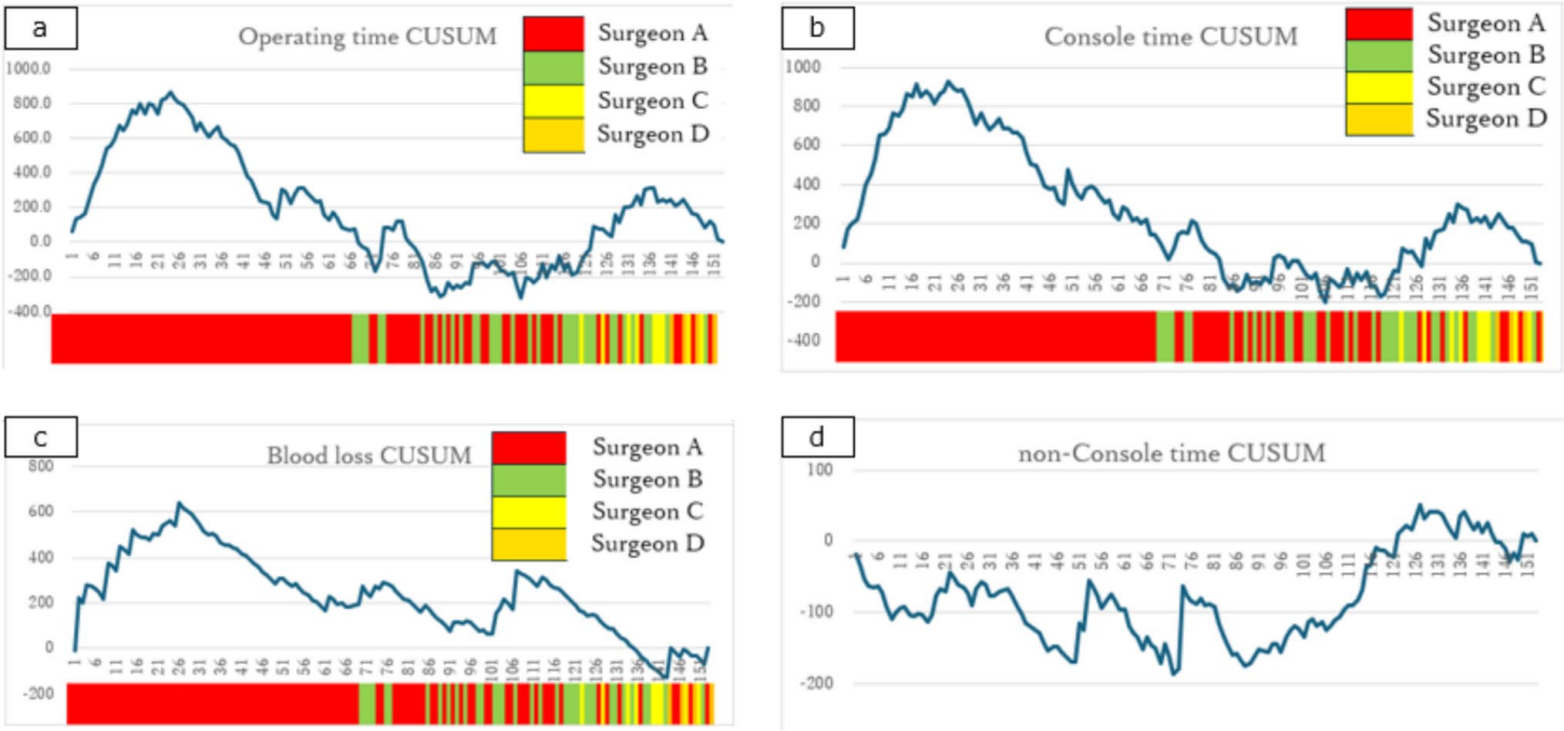
Cumulative sum (CUSUM) learning curves at our institution drawn using the cumulative sum method **a** Operating time. **b** Console time. **c** Blood loss. **d** non-Console time

The average operating time was 206.5 min, console time was 153.3 min, non-console time was 53.2 min, and blood loss was 23.9 g. In Fig. 2, each graph is supplemented with color-coded bars representing the individual surgeons. The operating and console times initially formed an upward convex learning curve for Surgeon A, followed by another upward convex curve as new surgeons joined the program. However, the peaks of the curves for subsequent surgeons were lower than those of the initial curve for Surgeon A (Fig. 2a, b).

The learning curve for blood loss was not affected by the inclusion of new surgeons (Fig. 2c). The non-console time initially followed a downward convex curve, and an upward trend was observed between the 88^th^ and 128^th^ cases (Fig. 2d).

**Fig. 3:**
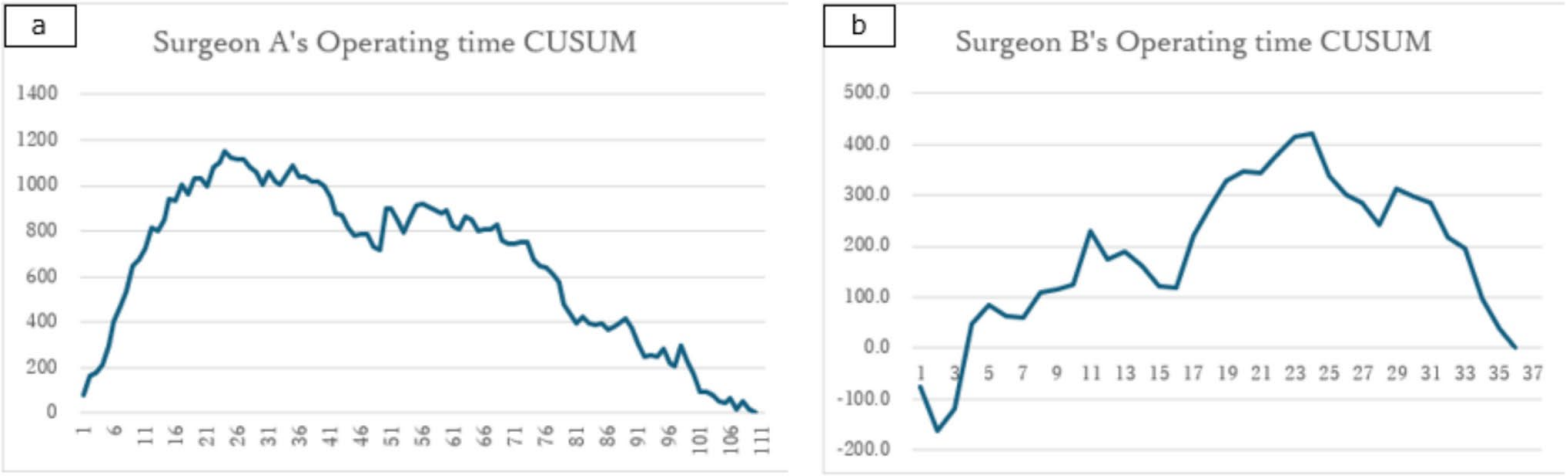
Learning curve of each surgeon drawn using the cumulative sum method. **a** Surgeon A. **b** Surgeon B. *CUSUM* cumulative sum

When the learning curves were analyzed individually for Surgeons A and B, Surgeon A exhibited a single prominent upward convex peak with a standard shape, and Surgeon B demonstrated a non-linear progression during the first 16 cases, followed by an upward convex curve (Fig. 3 a, b).

### Conversion to thoracotomy

None of the patients required a conversion to thoracotomy.

### Complications

Postoperative complications were observed in 13 patients (8.4%): atelectasis in 2 patients (1.2%), prolonged air leak in 2 patients (1.3%), arrhythmia in 1 patient (0.6%), pleurisy in 1 patient (0.6%), chylothorax in 1 patient (0.6%), pleural effusion in 1 patient (0.6%), urinary retention in 1 patient (0.6%), recurrent laryngeal nerve palsy in 1 patient (0.6%), post-dural puncture headache in one patient (0.6%), airway obstruction during extubation in 1 patient (0.6%), and cerebral infarction in 1 patient (0.6%). Mechanical dysfunction occurred in 11 cases (7.1%): 9 (5.8%) involved initial system malfunction, resolved by system reboot, and 2 (1.3%) involved temporary motion reversal. Among the 11 cases of mechanical malfunction, 4 occurred during procedures performed by Surgeon A between the 58th and 102nd cases, and 7 occurred during the early operative cases performed by Surgeon B between the 90th and 143rd cases. No mechanical malfunctions were recorded for Surgeons C and D.

## Discussion

The surgery and console time duration showed a regained upward trend when new surgeons joined. However, the peak of the curve is smaller compared with that at the initial phase, indicating an improvement in institutional proficiency. When Surgeon B joined the team, they alternated operative participation with Surgeon A, resulting in a minimal decline in proficiency. However, with the addition of Surgeon D, the participation of Surgeon A had already decreased, leading to a more pronounced upward trend in proficiency decline compared with the period following the inclusion of Surgeon B alone. Nevertheless, an improvement trend was observed in the later phase. This study showed that institutional proficiency did not significantly decline even with new surgeons, and the procedures could continue seamlessly. This indicates that the primary surgeons successfully conveyed their experiences, thoughts, and procedural outcomes. However, notably, the recipients of this knowledge already had a certain level of experience. A key challenge is transferring skills to junior surgeons who may perform their first anatomical lung resection using RATS.

This study showed educational challenges highlighted by atypical CUSUM curves. The primary surgeon A exhibited a typical CUSUM curve during the initial implementation phase. The second surgeon (B), whom the primary surgeon trained, demonstrated an atypical curve, forming one peak by the 15th case, followed by another larger peak. As the learning curve suggests, the initial implementation was remarkably smooth, allowing procedures to be performed without a mentor thereafter. However, this was simply due to scheduling constraints. This indicates the potential challenges in the educational approach. Thanks to the excellent operability of the robotic system, a second surgeon can complete the procedure efficiently under guidance. However, without supervision, they struggled to utilize their accumulated experience fully and spent significant time strategizing. Ideally, they should have learned key strategies while receiving guidance. However, the smooth operability of the robotic system may have led them to achieve independence before fully absorbing the theoretical framework. This phenomenon reflects the Dunning–Kruger effect [7], wherein beginners often exhibit overconfidence due to a one-dimensional understanding of their fields.

RATS has excellent operability, enabling surgeons to successfully complete procedures with minimal advice. This is attributed to features unique to robotic-assisted surgery, such as articulating instruments with wrist-like motions that improve maneuverability during complex procedures, including bronchial anastomosis. Additionally, motion scaling and tremor (quiver) elimination, which is not available in conventional VATS, supports a more accurate dissection [8, 9]. However, this ease of use may foster overconfidence and cause difficulties when surgeons must make independent decisions. In this study, no serious complications occurred; however, relying solely on individual aptitude may pose risks in the long term. To prevent such issues, teams must share basic strategies and protocols to manage unexpected challenges.

Sero et al. reported a failure rate of robotic-assisted laparoscopic cases of 0.38%, which included nine cases of patients suffering an injury. Our results included 7.1% initial system malfunction—higher than that reported in the literature—but no patient injury occurred. Several factors may explain the higher malfunction rate observed in our study. Most malfunctions occurred during the initial implementation period, when both surgeons and surgical assistants were still gaining familiarity with the robotic system's setup and troubleshooting processes. Additionally, staff turnover among surgical assistants may have further disrupted team consistency and contributed to procedural errors, leading to malfunctions. These observations highlight that team proficiency, especially among surgical assistants, plays a crucial role in minimizing technical problems. Establishing standardized training programs and rigorous troubleshooting protocols may be essential to further reduce system failures and maintain patient safety in robotic-assisted surgery. This means that, although surgeons experienced many issues due to unfamiliarity with the technique, most malfunctions occurred during implementation and did not cause direct harm to the patients [10].

Most malfunctions are related to setup issues, instrument problems, or system errors [11, 12]. In our study too, most failures were attributed to setup problems, which surgical assistants often resolved. These issues were also observed more frequently in later cases involving assistants with less surgical experience. Consequently, the non-console time showed an upward trend in later cases. This trend was likely attributable not only to transitions between surgeons but also to a decrease in surgical assistant experience due to staff turnover and limited robotic-specific training. Assistants unfamiliar with robotic setup and troubleshooting may have prolonged non-console processes, highlighting the importance of assistant-level proficiency in maintaining overall surgical efficiency. Notably, Surgeon A experienced no mechanical malfunctions during the first 50 cases, likely facilitated by the consistent support of experienced surgical assistants. In contrast, as the program expanded and less experienced assistants participated, system malfunctions increased. During the early operative periods of Surgeons C and D, strategic team management, including the assignment of experienced assistants as first assistants, likely contributed to maintaining a low rate of malfunctions. These findings suggest that maintaining a high level of assistant proficiency—particularly during phases of program expansion and new surgeon onboarding—is critical to sustaining surgical safety and efficiency. Improved design, enhanced safety protocols, and comprehensive training are recommended to reduce failures further and improve overall safety in robotic surgeries [13, 14].

Our findings are consistent with previous multi-institutional studies that emphasize the importance of standardized protocols and stepwise training in maintaining proficiency during RATS program expansion. Cerfolio et al. demonstrated that structured implementation strategies, including simulation training, supervised case progression, and team-based learning, significantly improved operative outcomes and minimized complication rates in robotic thoracic surgery programs [15].

These findings highlight the need to establish formal training programs that not only focus on console surgeons but also ensure comprehensive team-based system management. Future research should explore the development and validation of universal training frameworks applicable across different institutions.

This study has some limitations. As a single-center retrospective study with a limited number of cases and surgeons, there is a risk of selection and performance biases. While the current results appear favorable, an increase in the number of cases may lead to a higher incidence of complications. Moreover, as more surgeons participate, variations in surgical proficiency could impact the outcomes, potentially leading to less favorable results in a broader clinical setting. Therefore, further multicenter studies with a larger sample size are necessary to validate these findings.

## Conclusion

This study demonstrated that, while introducing new surgeons created fluctuations in performance metrics, overall institutional proficiency improved with the effective transfer of skills and strategies. Future studies should focus on refining training programs and assessing their impact across multiple institutions. The findings of this study provide insight into formulating approaches to enhance the quality of surgical care.

## Data Availability

All data supporting the findings of this study are available within the paper and its Supplementary Information.
